# Diagnostic accuracy of three-dimensional contrast-enhanced ultrasound for focal liver lesions: A protocol for systematic review and meta-analysis

**DOI:** 10.1097/MD.0000000000028147

**Published:** 2021-12-23

**Authors:** Meijng Qu, Zhaohua Jia, Lipeng Sun, Hui Wang

**Affiliations:** Ultrasound Department of the First Affiliated Hospital of Dalian Medical University.

**Keywords:** focal liver lesions, meta-analysis, three-dimensional contrast-enhanced ultrasound

## Abstract

**Background:**

Contrast-enhanced ultrasound (CEUS) examination is a well-established technique for this purpose with several unique advantages. It is a real-time technology with high temporal resolution. With its unique ability to detect microvascular perfusion, it helps in better characterization of FLL.^[1–4]^ Three-dimensional (3D) CEUS with quantitative analysis is updated in recent years. 3D-CEUS is a new ultrasonic diagnostic technique, which can observe the nourishing vessels of lesions from multiple angles. Previous studies showed that 3D-CEUS can detect tumor nourishing vessels to differentiate benign from malignant focal liver lesions (FLLs). However, the results of these studies have been contradictory. Therefore, this meta-analysis tested the hypothesis that 3D-CEUS is accurate in distinguishing benign and malignant FLLs.

**Methods:**

We will search PubMed, Web of Science, Cochrane Library, and Chinese biomedical databases from their inceptions to the April 30, 2021, without language restrictions. Two authors will independently carry out searching literature records, scanning titles and abstracts, full texts, collecting data, and assessing risk of bias. Review Manager 5.2 and Stata14.0 software will be used for data analysis.

**Results:**

This systematic review will determine the accuracy of 3D-CEUS in the differential diagnosis between benign and malignant FLLs.

**Conclusion:**

Its findings will provide helpful evidence for the accuracy of 3D-CEUS in the differential diagnosis between benign and malignant FLLs.

**Systematic review registration:**

INPLASY202150096.

## 1 Introduction

Hepatocellular carcinoma (HCC) is the most common primary hepatic malignancy and the third most common cause of cancer mortality globally and in the Asia-Pacific region.^[5]^ Once a liver nodule is identified on ultrasound, further radiological characterisation is typically warranted to elucidate the enhancement characteristics in order to determine the likelihood of HCC.^[6,7]^ Ultrasound scanning, along with other imaging technologies such as CT and MRI, is important in diagnosing and planning treatment for many people with liver disease. Contrast-enhanced ultrasonography (CEUS) is a safe, robust, and cost-effective imaging modality for evaluating focal liver lesions (FLLs) by depicting tumor vascularity in real time^[8–10]^. Computed tomography (CT) can also display the spatial structure of FLLs, but 3D-CEUS makes it possible to evaluate the response repeatedly during shortterm follow-up, without the use of ionizing radiation or potentially nephrotoxic contrast agents.^[11,12]^

3D-CEUS showed rapid enhancement of tumor trophoblastic vessels at the initial stage of the artery, and presented a “dendritic” distribution feature.^[12,13]^ The origin, course and spatial location of the trophoblastic vessels were clearly visible, which could better display the spatial location relationship between tumor trophoblastic vessels and neighboring vessels as well as the tumor body.^[7,14,15]^ 3D-CEUS can obtain the perfusion information of the entire tumor microvessels within a relatively short period of time, and the information obtained by it is relatively complete.^[3,16–19]^ Therefore, the evaluation of three-dimensional blood supply characteristics of tumors by 3D-CEUS plays an important role in the differential diagnosis of benign and malignant tumors.^[14,20]^ This work will systematically evaluate the technical performance and accuracy of 3D-CEUS for differential diagnosis of benign and malignant FLLs.

## 2 Materials and methods

This study was conducted in accordance with the preferred reporting items for systematic reviews and meta-analyses guidelines and the protocol was registered in the INPLASY (INPLASY202150096).

### 2.1 Eligibility criteria

#### 2.1.1 Type of study

This study will only include high quality clinical cohort or case control studies.

#### 2.1.2 Type of patients

The patients should be those who had undergone FLLs.

#### 2.1.3 Intervention and comparison

This study compare 3D-CEUS with pathology for diagnosing FLLs.

#### 2.1.4 Type of outcomes

The primary outcomes include sensitivity, specificity, positive and negative likelihood ratio, diagnostic odds ratio, and the area under the curve of the summary receiver operating characteristic.

### 2.2 Search methods

PubMed, Web of Science, Cochrane Library, and Chinese biomedical databases will be searched from their inceptions to the April 30, 2021, without language restrictions. The search strategy for PubMed is shown in Table 1. Other online databases will be used in the same strategy.

**Table 1:** Search strategy sample of pubmed.

Number	Search terms
1	Focal liver lesions or hepatic tumors or hepatocellular carcinoma
2	Three-dimensional contrast-enhanced ultrasound or three dimensional contrast enhanced ultrasound or 3D-CEUS
3	Pathology
4	And 1–3

3D-CEUS = three-dimensional contrast-enhanced ultrasound.

### 2.3 Data extraction and quality assessment

Two authors will independently select the trials according to the inclusion criteria, and import into Endnote X9 (Thomson Corporation, Stanford, USA). Then remove duplicated or ineligible studies. Screen the titles, abstracts, and full texts of all literature to identify eligible studies. All essential data will be extracted using previously created data collection sheet by 2 independent authors. Discrepancies in data collection between 2 authors will be settled down through discussion with the help of another author. The following data will be extracted from each included research: the first authors surname, publication year, language of publication, study design, sample size, number of lesions, source of the subjects, instrument, “gold standard”, and diagnostic accuracy. The true positives, true negatives, false positives, and false negatives in the fourfold (2 × 2) tables were also collected. Methodological quality was independently assessed by 2 researchers based on the quality assessment of studies of diagnostic accuracy studies (QUADAS) tool. The QUADAS criteria included 14 assessment items. Each of these items was scored as “yes” (2), “no” (0), or “unclear” (1). The QUADAS score ranged from 0 to 28, and a score ≥22 indicated good quality. Any disagreements between 2 investigators will be solved through discussion or consultation by a 3rd investigator.

### 2.4 Statistical analysis

The STATA version 14.0 (Stata Corp, College Station, TX) and Meta-Disc version 1.4 (Universidad Complutense, Madrid, Spain) softwares were used for meta-analysis. We calculated the pooled summary statistics for sensitivity, specificity, positive and negative likelihood ratio, and diagnostic odds ratio with their 95% confidence intervals. The summary receiver operating characteristic curve and corresponding area under the curve were obtained. The threshold effect was assessed using Spearman correlation coefficients. The Cochran Q-statistic and I test were used to evaluate potential heterogeneity between studies. If significant heterogeneity was detected (Q test *P* < .05 or I test > 50%), a random effects model or fixed effects model was used. We also performed sub group and meta-regression analyses to investigate potential sources of heterogeneity. To evaluate the influence of single studies on the overall estimate, a sensitivity analysis was performed. We conducted Begg funnel plots and Egger linear regression tests to investigate publication bias.

### 2.5 Ethics and dissemination

We will not obtain ethic documents because this study will be conducted based on the data of published literature. We expect to publish this study on a peer-reviewed journal.

## 3 Discussion

HCC is one of the most common cancers worldwide, and its accurate differentiation is important for clinical decision-making.^[5]^ The number of neovascularization is closely related to the growth mode, metastasis speed, and degree of tumor. There are also great differences in the number, structure, and spatial distribution of neovascularization in benign and malignant tumors.^[7,20]^ 3D-CEUS can display the origin location, course characteristics, number of branches and three-dimensional spatial distribution of the blood vessels in the lesion, and then make qualitative diagnosis of FLLS.^[14–17]^ This work will systematically evaluate the technical performance and accuracy of 3D-CEUS for differential diagnosis of benign and malignant FLLs.

### 3.1 Uncited references

^[1,2,4]^.

## Author contributions

**Conceptualization:** Lipeng Sun, Hui Wang.

**Data curation:** Meijing Qu, Zhaohua Jia.

**Methodology:** Meijing Qu, Zhaohua Jia.

**Writing – original draft:** Meijing Qu.

**Writing – review & editing:** Meijing Qu, Hui Wang.

## References

[1] Fang L Huang BJ Ding H. Contrast-enhanced ultrasound (CEUS) for the diagnosis of hypoechoic hepatic hemangioma in clinical practice. Clin Hemorheol Microcirc 2019;72:395–405. 30909196 10.3233/CH-190558

[2] Trillaud H Bruel JM Valette PJ. Characterization of focal liver lesions with SonoVue-enhanced sonography: international multicenter-study in comparison to CT and MRI. World J Gastroenterol 2009;15:3748–56. 19673015 10.3748/wjg.15.3748 PMC2726452

[3] Sasaki N Okumura Y Watanabe I. Relations between contact force, bipolar voltage amplitude, and mapping point distance from the left atrial surfaces of 3D ultrasound- and merged 3D CT-derived images: Implication for atrial fibrillation mapping and ablation. Heart Rhythm 2015;12:36–43. 25218838 10.1016/j.hrthm.2014.09.007

[4] Ge J Zhang X Zhang B. Higher tumor protein kinase D1 correlates with increased tumor size, BCLC stage, CA199 level, AFP level and worse overall survival in hepatocellular carcinoma patients. Clin Res Hepatol Gastroenterol 2021;45:101573. 33281070 10.1016/j.clinre.2020.11.004

[5] McGlynn KA Petrick JL London WT. Global epidemiology of hepatocellular carcinoma: an emphasis on demographic and regional variability. Clin Liver Dis 2015;19:223–38. 25921660 10.1016/j.cld.2015.01.001 PMC4712629

[6] Omata M Cheng AL Kokudo N. Asia-Pacific clinical practice guidelines on the management of hepatocellular carcinoma: a 2017 update. Hepatol Int 2017;11:317–70. 28620797 10.1007/s12072-017-9799-9 PMC5491694

[7] Marrero JA Kulik LM Sirlin CB. Diagnosis, staging, and management of hepatocellular carcinoma: 2018 practice guidance by the American Association for the study of liver diseases. Hepatology 2018;68:723–50. 29624699 10.1002/hep.29913

[8] Ferraioli G Meloni MF. Contrast-enhanced ultrasonography of the liver using SonoVue. Ultrasonography 2018;37:25–35. 28830058 10.14366/usg.17037 PMC5769944

[9] Bartolotta TV Taibbi A Midiri M Lagalla R. Contrast-enhanced ultrasound of hepatocellular carcinoma: where do we stand? Ultrasonography 2019;38:200–14. 31006227 10.14366/usg.18060 PMC6595127

[10] Bartolotta TV Taibbi A Picone D Anastasi A Midiri M Lagalla R. Detection of liver metastases in cancer patients with geographic fatty infiltration of the liver: the added value of contrast-enhanced sonography. Ultrasonography 2017;36:160–9. 28145108 10.14366/usg.16041 PMC5381848

[11] Yan J Schwartz LH Zhao B. Semiautomatic segmentation of liver metastases on volumetric CT images. Med Phys 2015;42:6283–93. 26520721 10.1118/1.4932365 PMC4600084

[12] Yamamichi J Kawaguchi Y Otsuka T. Assessment of tumor volume and density as a measure of the response of advanced hepatocellular carcinoma to sorafenib: application of automated measurements on computed tomography scans. JGH Open 2019;4:145–52. 32280757 10.1002/jgh3.12230 PMC7144795

[13] Dong FJ Xu JF Du D. 3D analysis is superior to 2D analysis for contrast-enhanced ultrasound in revealing vascularity in focal liver lesions – a retrospective analysis of 83 cases. Ultrasonics 2016;70:221–6. 27235776 10.1016/j.ultras.2016.05.007

[14] Cao J Dong Y Fan P Mao F Wang W. Feasibility of dynamic three-dimensional contrast-enhanced ultrasound in focal liver lesions: image quality evaluation and correlation of quantification with two-dimensional contrast-enhanced ultrasound. Clin Hemorheol Microcirc 2019;72:305–16. 30856104 10.3233/CH-180531

[15] Fang C Bernardo S Sellars ME Deganello A Sidhu PS. Contrast-enhanced ultrasound in the diagnosis of pediatric focal nodular hyperplasia and hepatic adenoma: interobserver reliability. Pediatr Radiol 2019;49:82–90. 30267165 10.1007/s00247-018-4250-5

[16] Lyshchik A Kono Y Dietrich CF. Contrast-enhanced ultrasound of the liver: technical and lexicon recommendations from the ACR CEUS LI-RADS working group. Abdom Radiol (NY) 2018;43:861–79. 29151131 10.1007/s00261-017-1392-0 PMC5886815

[17] Dietrich CF Nolsøe CP Barr RG Berzigotti A Burns PN. Guidelines and good clinical practice recommendations for contrast-enhanced ultrasound (CEUS) in the liver-update 2020 WFUMB in cooperation with EFSUMB, AFSUMB, AIUM, and FLAUS. Ultrasound Med Biol 2020;46:2579–604. 32713788 10.1016/j.ultrasmedbio.2020.04.030

[18] Schellhaas B Strobel D. Tips and tricks in contrast-enhanced ultrasound (CEUS) for the characterization and detection of liver malignancies. Ultraschall Med 2019;40:404–24. 31382313 10.1055/a-0900-3962

[19] Kong WT Wang WP Huang BJ Ding H Mao F Si Q. Contrast-enhanced ultrasound in combination with color Doppler ultrasound can improve the diagnostic performance of focal nodular hyperplasia and hepatocellular adenoma. Ultrasound Med Biol 2015;41:944–51. 25701530 10.1016/j.ultrasmedbio.2014.11.012

[20] Zarzour JG Porter KK Tchelepi H Robbin ML. Contrast-enhanced ultrasound of benign liver lesions. Abdom Radiol (NY) 2018;43:848–60. 29167944 10.1007/s00261-017-1402-2

